# Development of an adoptive cell therapy protocol with tumor-infiltrating lymphocytes and intermediate-dose interleukin-2 therapy

**DOI:** 10.1186/2051-1426-1-S1-P25

**Published:** 2013-11-07

**Authors:** Linh T Nguyen, Marcus O Butler, Pei Hua Yen, Jessica Nie, Michael Pniak, Alisha R Elford, Anthony M Joshua, David Hogg, Danny Ghazarian, Ayman Al-Habeeb, Alexandra M Easson, Wey L Leong, David R McCready, Michael Reedijk, Hans A Messner, Pamela S Ohashi

**Affiliations:** 1 Campbell Family Institute for Breast Cancer Research, Princess Margaret Cancer Centre, University Health Network, Toronto, ON, Canada; 2 Department of Medical Oncology and Hematology, Princess Margaret Cancer Centre, University Health Network, Toronto, ON, Canada; 3 Department of Pathology, University Health Network, Toronto, ON, Canada; 4 Department of Surgical Oncology, Princess Margaret Cancer Centre, University Health Network, Toronto, ON, Canada; 5 Department of Immunology, University of Toronto, Toronto, ON, Canada

## 

Adoptive cell therapy (ACT) with tumor-infiltrating lymphocytes (TILs) has shown promising results in early phase trials. In an approach developed by the Rosenberg group at the Surgery Branch (NCI), patients with metastatic melanoma receive a pre-conditioning regimen of cyclophosphamide and fludarabine-induced lymphodepletion (+/- total body irradiation), followed by infusion of autologous TILs and high-dose interleukin-2 (IL-2) therapy. At our institution, we have established the technology to produce therapeutic-grade TILs. Our preclinical work included adapting protocols from the Surgery Branch to expand TILs from melanoma lesions, setting up a program to obtain blood products from healthy donors to support the in vitro expansion of TILs, and establishing Standard Operating Procedures for production of therapeutic-grade TILs. In order to generate sufficient numbers of cells for infusion, TILs are subjected to a “Rapid Expansion Protocol” (REP) using anti-CD3 antibody (OKT3), feeder cells (irradiated peripheral blood mononuclear cells) and interleukin-2. In our preclinical work, we found that the REP could be performed using concentrations of IL-2 that are lower than those usually used: 300 and 600 IU/ml of IL-2 induced similar fold-expansion and CD4:CD8 ratios as 3000 IU/ml of IL-2. Survival of TILs in vitro after the REP in various concentrations of IL-2 was also assessed. We reasoned that TILs that had been rapidly expanded in a lower concentration of IL-2 would subsequently not require high-dose IL-2 after infusion in order to mediate clinical responses. If this is the case, use of a lower dose of IL-2 therapy for TIL protocols would improve the toxicity profile of ACT and reduce resources needed for inpatient care. In order to explore this hypothesis, we have designed a clinical trial of cyclophosphamide and fludarabine followed by TIL infusion and an intermediate dose of IL-2 therapy (125,000 IU/kg for 2 weeks with 2 days rest in between each week). Institutional and federal regulatory approvals have been obtained for this trial.

